# Vagus Nerve Stimulation Amplifies Task-Induced Cerebral Blood Flow Increase

**DOI:** 10.3389/fnhum.2021.726087

**Published:** 2021-08-09

**Authors:** Naoto Kunii, Tomoyuki Koizumi, Kensuke Kawai, Seijiro Shimada, Nobuhito Saito

**Affiliations:** ^1^Department of Neurosurgery, The University of Tokyo Hospital, Tokyo, Japan; ^2^Department of Neurosurgery, Jichi Medical University, Shimotsuke, Japan

**Keywords:** vagus nerve stimulation, cerebral blood flow, near-infrared spectroscopy, epilepsy, rehabilitation

## Abstract

**Background:**

Vagus nerve stimulation (VNS) is an established palliative surgical treatment for refractory epilepsy. Recently, pairing VNS with rehabilitation received growing attention for their joint effect on neural plasticity. However, objective biological measurements proving the interaction between VNS effects and cortical recruitment are lacking. Studies reported that VNS induced little blood flow increase in the cerebral cortex.

**Objective:**

This study tested the hypothesis that pairing VNS with a cognitive task amplifies task-induced cerebral blood flow (CBF).

**Methods:**

This study included 21 patients implanted with vagus nerve stimulator to treat refractory epilepsy. Near-infrared spectroscopy (NIRS) with sensors on the forehead measured CBF changes in the frontal cortices in response to VNS. Cerebral blood flow was measured when VNS was delivered during a resting state or a verbal fluency task. We analyzed the VNS effect on CBF in relation to stimulation intensity and clinical responsiveness.

**Results:**

We observed no CBF change when VNS was delivered during rest, irrespective of stimulation intensity or responsiveness. Cerebral blood flow changed significantly when a verbal fluency task was paired with VNS in a stimulation intensity-dependent manner. Cerebral blood flow changes in the non-responders showed no intensity-dependency.

**Conclusion:**

Our results could be an important biological proof of the interaction between VNS effects and cortical recruitment, supporting the validity of pairing VNS with rehabilitation.

## Introduction

Multiple studies have established the usefulness of vagus nerve stimulation (VNS) as a palliative surgical treatment for refractory epilepsy due to its seizure inhibition effect (Ben-Menachem et al., 1994; Ramsay et al., 1994; DeGiorgio et al., 2000; Helmers et al., 2001; Janszky et al., 2005; Elliott et al., 2011; Kawai et al., 2017). Interest in the effects of VNS on physiological and pathological conditions other than epilepsy has also been growing (Yuan and Silberstein, 2016). Among them, the VNS effect on the generation of neural plasticity has a significant impact. Engineer et al. (2011) reported that pairing a tone with VNS increased the proportion of the primary auditory cortex responding to the tone. Similarly, pairing VNS with a specific movement resulted in the recruitment of a larger cortical area corresponding to the movement, suggesting that this could be a general method for increasing cortical representation of specific functions (Porter et al., 2012). Behaviorally, pairing VNS with a movement task was shown to improve movement dysfunction after chronic stroke in animals and humans (Khodaparast et al., 2016; Kimberley et al., 2018). Thus, VNS is expected to be utilized in neurorehabilitation, and a pivotal study on such utilization is ongoing (Dawson et al., 2020).

Such temporally precise interactions between VNS and specific cortical functions suggest that some form of VNS-induced changes occur in the cerebral cortex. However, to date, no objective biological measurement proved such an interaction. Cerebral blood flow (CBF) is one of the most robust biological measures of brain activity induced by cognitive/behavioral tasks. Previous CBF studies have evaluated changes following VNS without any associated tasks and observed no increase in the cerebral cortex blood flow (Ko et al., 1996; Ring et al., 2000; Van Laere et al., 2002; Henry et al., 2004). Additionally, due to the limited time resolution of CBF studies, most studies focused on the chronic effects of VNS, providing little information about its immediate effects on the cerebral cortex.

In this study, we hypothesized that pairing VNS with a task would amplify the task-induced CBF. We used near-infrared spectroscopy (NIRS) to measure CBF during VNS paired or not with a cognitive task to test this hypothesis. NIRS can evaluate CBF by quantifying the concentration of different hemoglobin states by using near-infrared light at two optical path lengths (Villringer et al., 1993). It has a millisecond-level time resolution, making it superior to positron emission tomography and single-photon emission computed tomography. NIRS has been widely used to detect CBF changes during cognitive tasks (Watanabe et al., 1998), and seems to be the best way to investigate immediate CBF changes during VNS. We expected CBF to be impacted differently when VNS was paired with a cognitive task or not. Such a finding will strongly support the scientific validity of pairing VNS with rehabilitation and provide important clues to elucidate the still elusive mechanism of VNS as a treatment for epilepsy.

## Materials and Methods

### Participants

We recruited patients who underwent VNS system implantation as a surgical treatment for refractory epilepsy and were on regular follow-up at the University of Tokyo Hospital between August 2014 and July 2015. The inclusion criteria were as follows: age 18 and above, at least 6 months of VNS treatment history, current VNS intensity of 0.75 mA or more, native Japanese speaker, and being able to remain motionless during the measurements.

Basically, we increased VNS intensity by 0.25 mA every 3-month visit starting from 0.25 mA. Since VNS intensity is gradually increased to a therapeutic range (usually 1.5–3.0 mA), the current dose of each patient varied depending on the time after implantation and the severity of side effects of stimulation.

Based on the medical records and detailed information from the patients and their families, the participants were divided into responder and non-responder groups to evaluate the results in terms of long-term seizure outcome. Responders were patients whose seizure frequency decreased by 50% or more from the level during the last year before VNS implantation. Non-responders were patients with poorer outcomes than the criteria of responders. All participants underwent NIRS measurements when VNS was delivered to at rest (rVNS, see below for details). Before VNS implantation, patients were usually evaluated using Wechsler Adult Intelligence Scale-III in the preoperative workup. Patients showing verbal IQ > 65 also underwent NIRS measurement when VNS was delivered while performing a task (tVNS, see below for details).

The Institutional Review Board of the University of Tokyo approved this study (10501). Written informed consent was obtained from all patients or their families after presenting a detailed explanation of the study.

### Near-Infrared Spectroscopy

Near-infrared spectroscopy can estimate *in vivo* changes in CBF from variation in the hemoglobin concentration, utilizing the near-infrared light characteristics. NIRS is useful in non-invasively localizing functional changes in the brain with a millisecond-level time resolution. Unlike functional MRI, NIRS can be safely and conveniently applied to patients implanted with a VNS system. NIRS is, therefore, compatible with measuring CBF changes caused by VNS.

Measurements were performed with a NIRS system (ETG-4000, Hitachi Medical Corp., Tokyo, Japan). Fifty-two channels were measured with a 3 × 11 probe holder that covered both sides of the forehead. The distances between probes were 3.0 cm. The lowest row of probes was arranged on the T3-Fpz-T4 line of the international 10–20 system used in electroencephalography recording (Okamoto et al., 2004). Oxy- and deoxyhemoglobin were measured by near-infrared light at 695 and 830 nm, respectively. We focused on analyzing oxyhemoglobin, which was reported to be very well associated with the cerebral cortex activity (Hoshi et al., 2001), and has been used as a valuable biomarker in many studies. Hemoglobin signal was expressed as the product of the change in the hemoglobin concentration (mM) and the optical pathlength (mm). The sampling frequency was 10 Hz.

### Measurement Environment and Task Paradigm

Hemoglobin signal was measured by NIRS in a quiet room, with the patient seated in front of a monitor and looking at a fixation point on it. We confirmed that there had been no seizures during the 2 h before each measurement. Antiepileptic drugs were taken as usual, and the VNS settings remained unchanged until immediately before the measurements.

**FIGURE 1:**
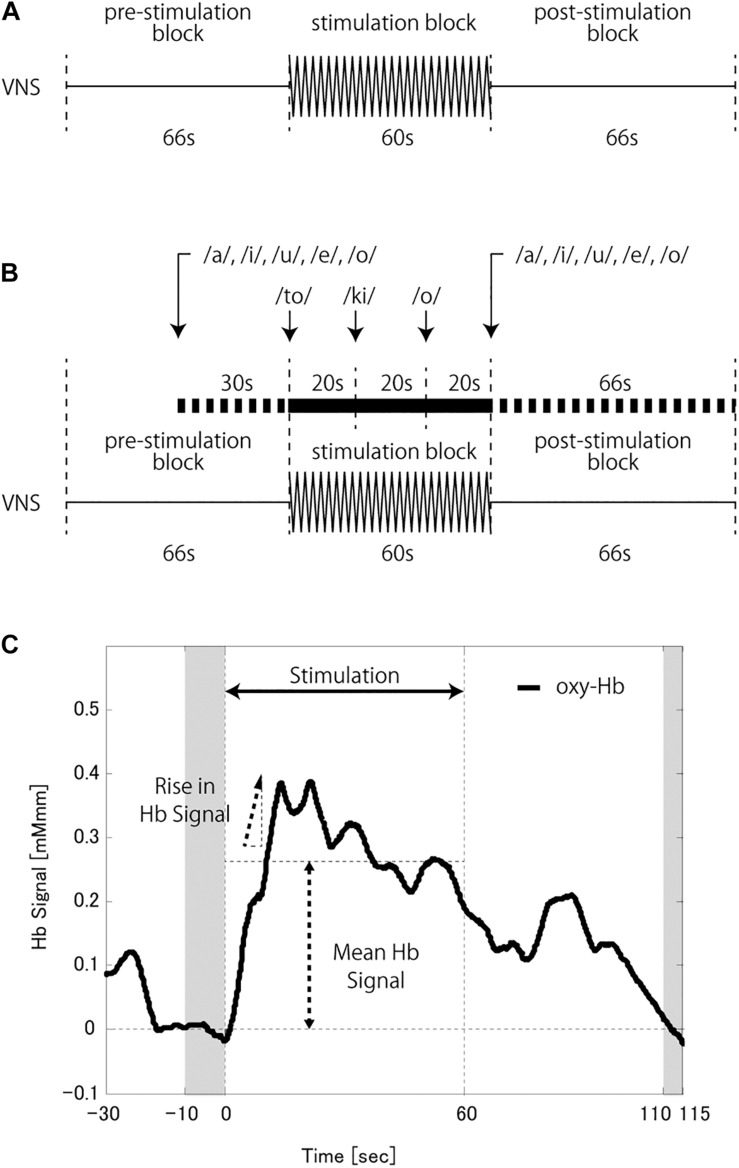
**(A)** Schema of the vagus nerve stimulation (VNS) at a rest (rVNS) condition. **(B)** Schema of the VNS at a task (tVNS) condition. **(C)** A schema of the hemoglobin (Hb) signal analysis. The baseline was corrected based on the average Hb signal at the last 10 s of the pre-stimulation block and the 5 s from 50 s to 55 s of the post-stimulation block (shaded time slots). The mean Hb signal was calculated as the signal mean during the stimulation block. The rise in Hb signal was defined as the peak change in Hb signal value per second during the first half of the stimulation block.

The measurement paradigm was designed to consist of two conditions, each of three blocks in the following order: pre-stimulation (66 s), stimulation (60 s), and post-stimulation (66 s). The VNS system was set to deliver stimulations throughout the stimulation block, during which the patient stayed at rest (rVNS condition; Figure 1A) or performed a verbal fluency task (tVNS condition; Figure 1B). The verbal fluency was a word generation task known to induce CBF in the frontal lobe effectively and is widely used during NIRS to support a depressive disorder diagnosis (Takizawa et al., 2014; Ho et al., 2020; Husain et al., 2020). The patients were instructed to vocally generate as many words as possible, beginning with each of the initial syllables presented. Syllables were presented through a speaker three times, once every 20 s. During tVNS, the patients were also asked to repeatedly utter the five Japanese vowels (a, i, u, e, o) during the last 30 s of the pre- stimulation block and the 66 s of the post-stimulation block. The CBF measurements during these periods were used as the baselines for the following analysis.

Patients with verbal IQ > 65 underwent measurements in tVNS and rVNS conditions. The VNS current intensity was set at treatment dose, then at a half dose (or near half dose when the treatment dose was indivisible), and finally at a zero dose. At zero dose, only measurements during tVNS were conducted. Patients with lower verbal IQ underwent rVNS measurement at the treatment dose and then at a half dose. We set a 3-min interval between measurements, during which no VNS stimulations were delivered.

The VNS on and off times were set to 66 and 60 s, respectively, to match the task paradigm. The VNS stimulation timing was synchronized with the stimulation block by the magnet and the normal mode of the VNS system. The pulse width and frequency were the same as used for each patient in the clinical settings.

### Data Analysis

The verbal fluency task is widely used during NIRS measurement to activate the frontal lobe effectively. We used data from the 11 channels on the forehead out of 52 channels, following previous studies (Takizawa et al., 2014; Husain et al., 2020). We used those channels to minimize the effect of frontotemporal craniotomy performed before VNS implantation. As described above, the oxyhemoglobin waveform of each channel was used as a biomarker of cerebral cortex blood flow.

Noise reduction and baseline correction were performed as preprocessing procedures. High-frequency noise, most of which was motion artifacts, was removed from the measured waveforms of the hemoglobin signal by the moving average method (the moving average window was 5 s) in the software embedded in the NIRS machine. The resulting data were analyzed by MATLAB (The MathWorks, Natick, MA, United States). The baseline was corrected by the least-square method, using the averaged hemoglobin signal of the last 10 s during the pre-stimulation block and the 5 s from 50 to 55 s of the post-stimulation block.

The average hemoglobin signal of the last 10 s of the pre-stimulation block was compared to the average hemoglobin signal during the stimulation block to identify CBF change. The mean hemoglobin signal (mHbS) and rise in the hemoglobin signal (rHbS) were calculated as indices of CBF change. mHbS was calculated as the mean hemoglobin signal during the stimulation block (Figure 1C). rHbS was the peak change in the hemoglobin signal value per second during the first half of the stimulation block after applying a moving average window of ten time points.

Demographic data were compared by the chi-squared test or Student’s *t*-test with a significance level of *p* < 0.05. All hemoglobin signal comparisons were channel-based and performed using Wilcoxon rank sum test with a significance level set at *p* < 0.05 (two-tailed). Three measurements from the same patients were compared by repeated-measures ANOVA, and Wilcoxon rank sum tests were used in the *post hoc* analysis. The false discovery rate (FDR) method was used to correct for multiple comparisons. All tests were two-tailed. Statistical analysis was performed using the JMP Pro, Version 14 (SAS Institute Inc., Cary, NC, United States).

## Results

### Participants Characteristics

Twenty-one patients (nine male and 12 female) were included in this study. Eight and 13 patients were responders and non-responders, respectively. All of them underwent NIRS measurement in rVNS condition. The mean age was 35.2 years (range, 18–51). The mean age at VNS implantation was 31.9 years (range, 12–52 years). Both age measures differed significantly between responders and non-responders. The mean VNS treatment duration was 39.5 months (range, 9–107 months), and the mean VNS output current was 1.67 mA (range, 0.75–2.75 mA). VNS was selected as the initial surgical treatment for 12 patients because of the multifocal nature of their epilepsy. Nine patients underwent VNS implantation as an additional surgical treatment after craniotomy had failed. Of these nine patients, eight underwent frontotemporal craniotomy, and one was treated by parietal craniotomy.

Twelve subjects who showed VIQ > 65 also underwent NIRS measurement in tVNS condition. Of the 12 subjects, five were responders and seven were non-responders. No differences were found between the two groups, except for age at epilepsy onset. Tables 1, 2 shows the demographic data for responders and non-responders treated by rVNS and tVNS, respectively.

**TABLE 1:** Demographic data for VNS at rest condition.

	**Responder (*n* = 8)**	**Non-responder (*n* = 13)**	***p*-value**
Age, year			0.0275^b^
Mean (range)	30 (18–43)	39 (29–51)	
Sex, *n* (%)			1.00^a^
Male	5 (62.5)	7 (53.8)	
Female	3 (37.5)	6 (46.2)	
Age at onset, year			0.225^b^
Mean (range)	11 (0–25)	16 (0–32)	
Age at implantation, year			0.0405^b^
Mean (range)	26 (12–43)	36 (21–50)	
Duration of VNS treatment, month			
Mean (range)	45 (9–103)	36 (10–107)	0.523^b^
Output current, mA			
Mean (range)	1.75 (1.00–2.75)	1.62 (0.75–2.50)	0.623^b^
Verbal IQ			
Mean (range)	70 (50–96)	77 (47–94)^*c*^	0.367^b^
Previous epilepsy surgery, *n* (%)	2 (25.0)	7 (53.8)	0.367^a^

*^*a*^Chi-squared test.*

*^*b*^Two-tailed *t*-test.*

*^*c*^Data were not available for three subjects.*

*VNS, vagus nerve stimulation.*

**TABLE 2:** Demographic data for VNS at task condition.

	**Responder (*n* = 5)**	**Non-responder (*n* = 7)**	***p*-value**
Age, year			0.0906^b^
Mean (range)	31 (19–43)	41 (29–51)	
Sex, *n* (%)			0.293^a^
Male	1 (20.0)	4 (57.1)	
Female	4 (80.0)	3 (42.9)	
Age at onset, year			0.0106^b^
Mean (range)	10 (2–18)	22 (15–32)	
Age at implantation, year			0.0625^b^
Mean (range)	26 (12–43)	39 (28–50)	
Duration of VNS treatment, month			
Mean (range)	53 (9–103)	26 (10–48)	0.123^b^
Output current, mA			
Mean (range)	1.75 (1.00–2.00)	1.43 (0.75–2.50)	0.365^b^
Verbal IQ			
Mean (range)	80 (71–96)	83 (69–94)	0.551^b^
Previous epilepsy surgery, *n* (%)	1 (20.0)	3 (42.9)	0.576^a^

*^*a*^Chi-squared test.*

*^*b*^Two-tailed *t*-test.*

*VNS, vagus nerve stimulation.*

### Cerebral Blood Flow Change During the rVNS

All 21 patients completed the rVNS, of which eight were responders, and 13 were non-responders.

**FIGURE 2:**
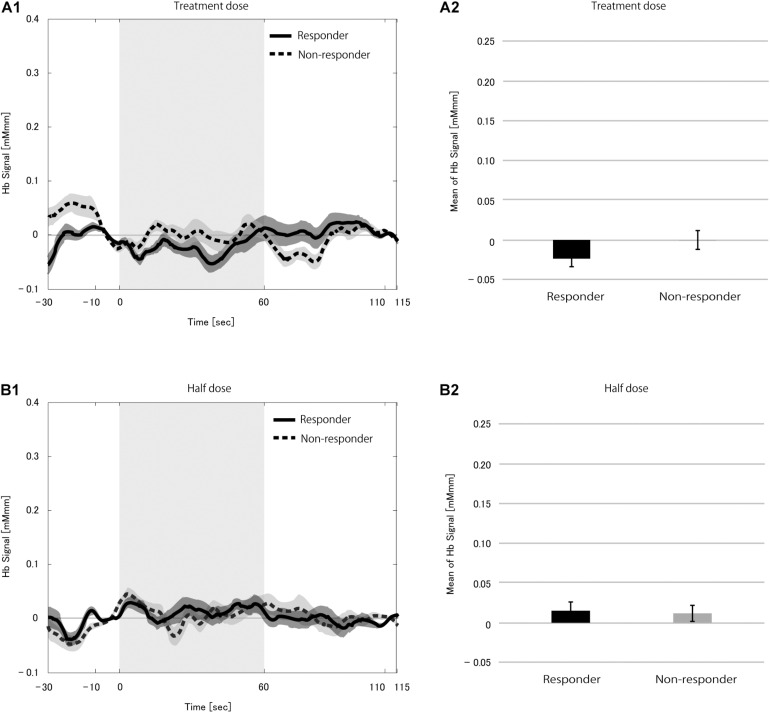
Grand average of the change in hemoglobin (Hb) signal **(A1,B1)** and mean Hb signal **(A2,B2)** in the 21 patients during the vagus nerve stimulation (VNS) at rest condition at the treatment dose **(A)** and the half dose **(B)**. Solid and dotted line indicate responders and non-responders, respectively. The shaded area around each line indicates the standard error of the mean values. Gray-shaded periods indicate the stimulation block during which the VNS stimulation was on. There was no difference in mean Hb signal between responders and non-responders at either dose.

We found no difference in the hemoglobin signal between the stimulation and pre-stimulation blocks during rVNS measurements (treatment dose, *p* = 0.289; half dose, *p* = 0.103). We compared mHbS between responders and non-responders at each dose to rule out the possibility that difference in the immediate VNS effect on the brain at rest masked changes in the CBF, but found no difference at both dose levels (treatment dose, *p* = 0.090; half dose, *p* = 0.939; Figure 2).

### Cerebral Blood Flow Changes During the tVNS

Twelve patients completed the tVNS, of which five were responders, and seven were non-responders.

**FIGURE 3:**
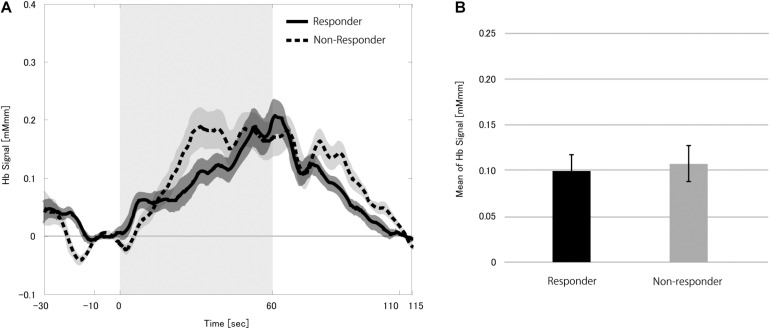
Grand average of the change in the hemoglobin (Hb) signal **(A)** and mean Hb signal **(B)** in the 12 patients during zero dose vagus nerve stimulation (VNS) at the task condition. Solid and dotted lines indicate responders and non-responders, respectively. The shaded area around each line indicates standard error of the mean values. Gray-shaded period indicates the stimulation block even though the VNS dose was set to zero. The mean Hb signals in the responders and non-responders were similar.

We first studied hemoglobin signal changes during the stimulation block when performing the verbal fluency task without VNS to find if CBF changes were induced purely due to the task. Although we found a significant increase in the hemoglobin signals (*p* < 0.0001), the mHbSs in the responders and non-responders were similar (*p* = 0.411; Figure 3). These results indicated that the verbal fluency task effectively induced an increase in the CBF. The VNS effect described below could not be attributable to the difference in responsiveness to the verbal fluency task between responders and non-responders.

Next, we compared mHbSs at different VNS doses between responders and non-responders to verify the effect of VNS on CBF changes. The change in CBF was significantly larger in responders than in non-responders at the treatment dose (*p* < 0.0001), but not at the half dose (*p* = 0.092).

**FIGURE 4:**
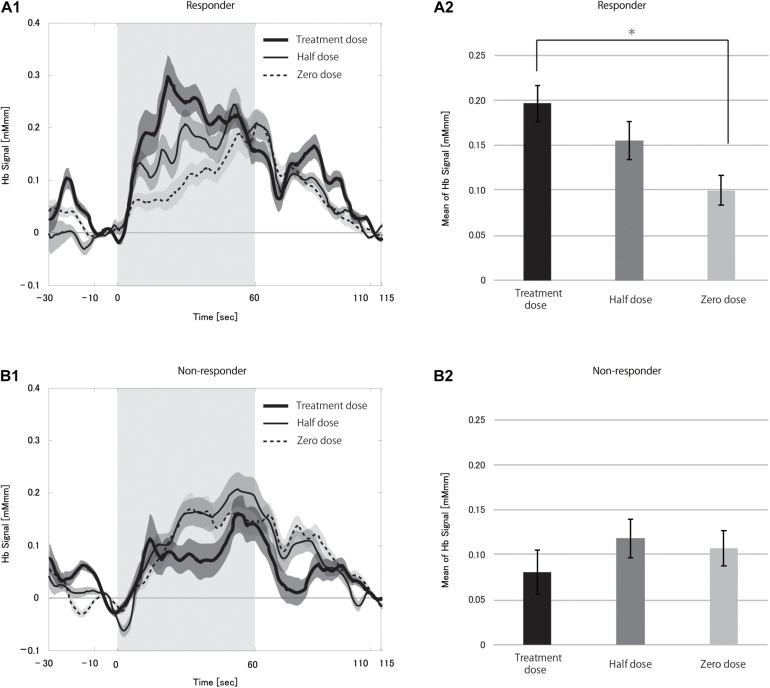
Grand averages of the hemoglobin (Hb) signal **(A1,B1)** and mean Hb signal **(A2,B2)** in responders **(A)** and non-responders **(B)** during the vagus nerve stimulation (VNS) at the task condition. In **A1,B1**, thick solid, thin solid and dotted lines indicate treatment dose, half dose, and zero dose, respectively. The shaded area around each line indicates the standard error of the mean values. Gray-shaded periods indicate the stimulation block. The mean Hb signal differed significantly between treatment and zero dose in responders (*p < 0.05). There were no differences between the doses in the non-responders.

We compared the hemoglobin signals between different VNS doses separately in responders and non-responders to clarify the CBF dose-dependency on VNS in this paradigm (Figure 4, see Supplementary Figure 1 for box-whisker plot version of this figure with each data point). Repeated-measures ANOVA revealed a significant difference between the three dose levels in responders (*p* < 0.0001), but not in non-responders (*p* = 0.163). *Post hoc* analysis found the treatment dose to be significantly different from the zero dose after correction for multiple comparisons (*p* = 0.0024). Although the difference between half and zero dose was not significant, there was a tendency where the higher the current output, the larger the CBF change. This strongly supported the notion that VNS could have an immediate effect on CBF change induced by a cognitive task.

**FIGURE 5:**
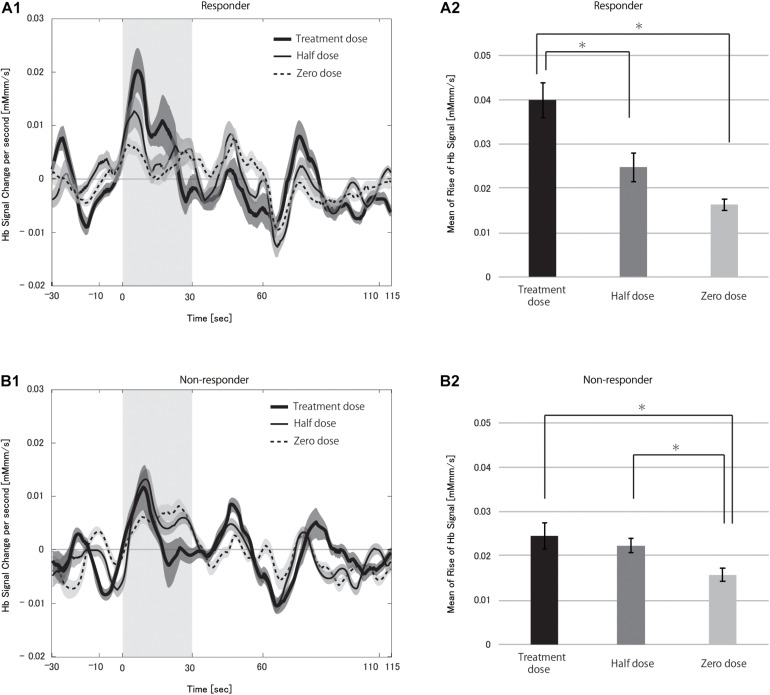
Grand averages of the change in the hemoglobin (Hb) signal per second **(A1,B1)** and mean rise in Hb signal **(A2,B2)** in responders **(A)** and non-responders **(B)** during the vagus nerve stimulation (VNS) at the task condition. In **A1,B1**, thick solid, thin solid, and dotted lines indicate treatment dose, half dose, and zero dose, respectively. The shaded area around each line indicates the standard error of the mean values. Gray-shaded periods indicate the time range during which the rise in Hb signal was analyzed. We found significant differences in the mean rise in Hb signal between treatment and half doses and treatment and zero doses in responders (*p < 0.05 for both). There were also significant differences between treatment and zero doses and half and zero doses in non-responders (*p < 0.05).

The time course of hemoglobin signals of responders in Figure 5 demonstrated the dose-dependency in the degree of CBF change and suggested that the presence of differences in the hemoglobin signal initial increases among doses. Thus, we examined the rHbSs. First, we compared rHbSs between responders and non-responders at each VNS dose. At the treatment dose, the rHbS was significantly higher in responders than in non-responders (*p* < 0.0001), but not at the half (*p* = 0.561) or zero (*p* = 0.218) doses. We then compared the rHbSs at the VNS doses, separately in responders and non-responders, to study VNS dose-dependency. Repeated-measures ANOVA revealed significant differences in responders (*p* < 0.0001) and non-responders (*p* < 0.0001; Figure 5, see Supplementary Figure 2 for box-whisker plot version of this figure with each data point). *Post hoc* analysis found significant differences between treatment and half dose (*p* < 0.0001) and treatment and zero dose (*p* < 0.0001) in the responders. Treatment and zero dose (*p* = 0.002) and half and zero dose (*p* = 0.003) differed significantly in the non-responders.

## Discussion

We used NIRS to investigate changes in the CBF caused by VNS paired or not with a cognitive task, focusing on VNS immediate effects in patients at the chronic phase of VNS treatment. Our results showed that VNS did not alter the CBF at rest but amplified the CBF increase when accompanied by a cognitive task in VNS-responders. Importantly, this VNS effect was dose-dependent, supporting the conclusion that VNS caused the amplification of CBF increase. This is the first report to demonstrate biological evidence for dose-dependent brain modulation by pairing VNS with a cognitive/behavioral task. This result supports the validity of pairing VNS with rehabilitation and contributes to elucidating its mechanism.

Vagus nerve stimulation alone did not cause a change in CBF in the present study, neither in responders nor in non-responders. This finding is in line with the absence of studies reporting cerebral cortex CBF increase due to VNS. Although the verbal fluency task alone induced a CBF increase in responders and non-responders, the increase in both was similar. These findings show that the responders and non-responders responded similarly to either VNS or the verbal fluency task alone. Nevertheless, pairing VNS with the verbal fluency task amplified the CBF increase, but only in responders. This finding suggests that synchronized administration of VNS and a cognitive task is essential to facilitate cortical activation and that there is a common mechanism between CBF modulation and the anti-epileptic effect of VNS.

Various studies on the mechanism of the anti-epileptic effect of VNS have been conducted. EEG studies on the mechanism of VNS have a long history, in which animal studies have revealed desynchronization in the cerebral cortices (Magnes et al., 1961; Chase et al., 1967). Such cortical desynchronization has also been shown in human studies and was associated with the anti-epileptic effect of VNS (Nemeroff et al., 2006; Jaseja, 2010; Fraschini et al., 2013; Bodin et al., 2015). Besides, many CBF studies have been conducted in humans. In positron emission tomography studies, VNS-induced CBF increases were localized to the subcortical regions, including the thalamus (Ko et al., 1996; Henry et al., 2004). In contrast, single-photon emission computed tomography studies reported a decreased CBF in the thalamus (Ring et al., 2000; Van Laere et al., 2002). Although they were not necessarily consistent, most CBF studies have suggested that the thalamus is a key structure in the anti-epileptic effect of VNS. There have been several functional MRI studies on the VNS mechanism in the early days, before VNS was contraindicated for use in the MRI machine (Narayanan et al., 2002; Sucholeiki et al., 2002; Liu et al., 2003). These studies showed increased activity in the thalamus and cerebral cortices, in line with most CBF studies. Thus, it is assumed that VNS ameliorates epileptic seizures by causing cortical desynchronization through the thalamocortical network (Jaseja, 2010).

On the other hand, it is known that electrocorticographic spectral analysis shows cognitive/behavioral task-activated alpha-beta desynchronization in the cortex (Crone et al., 1998; Hirata et al., 2009), which is considered to reflect an engagement of the thalamocortical network (Neuper and Pfurtscheller, 2001). Considering the assumed mechanism of VNS anti-epileptic effect and the cortical activation observed as EEG desynchronization during a cognitive/behavioral task, it seems that VNS and the verbal fluency task in the present study acted simultaneously on the thalamocortical network and synergistically enhanced the cortical activity. The repetitive reinforcement of the thalamocortical network by pairing VNS with a specific task could elevate the responsiveness of the corresponding cortex, which might be the mechanism behind the effect of pairing VNS with rehabilitation. We demonstrated with NIRS that paired VNS, and the verbal fluency task increased the CBF more than the task alone, an important biological finding showing indirectly that VNS generates plasticity.

The dose-dependency of CBF observed in this study suggests various possibilities. First, the anti-epileptic effect of VNS is known to be dose-dependent (Ben-Menachem et al., 1994; Handforth et al., 1998). This similarity supports a common mechanism in VNS effects on CBF and epilepsy, as described above. Second, VNS is used to treat major depressive disorder, also with a dose-dependent effect (Aaronson et al., 2013). The CBF, when performing a verbal fluency task, was reported to be reduced in patients with major depressive disorder (Takizawa et al., 2014; Ho et al., 2020; Husain et al., 2020). Therefore, the improvement we observed in CBF responsiveness seems to share the same mechanism with the VNS effect on major depressive disorder. Third, we demonstrated that the time of CBF increase was also dose-dependent. The higher the dose, the faster the CBF increased. Porter et al. (2012) demonstrated the importance of temporal precision in paring VNS with a task for enhancing cortical plasticity. Therefore, the dose-dependency of the time of CBF increase found in this study suggests that precise timing of VNS and the task and a high enough VNS dose are necessary for efficient coupling between VNS and rehabilitation.

Finally, it should be noted that epileptic foci are known to have elevated levels of plasticity, which is characterized by (a) increments in the efficacy of synaptic transmission in pre-existing synapses; (b) induction of new synaptic connections and reordering of pre-existing contacts; and (c) improvement of the ability of neurons to become excited (Jarero-Basulto et al., 2018). In epileptic foci, these neuroplastic changes are thought to contribute to abnormal restructuring of the cortical functions. In the present study, we demonstrated dose-dependent CBF modulation in responders and assumed a common mechanism between CBF modulation and the anti-epileptic effect of VNS. From the standpoint of higher plasticity in epileptic foci, it could also be interpreted that anti-epileptic effect of VNS had optimized cortical plasticity in responders before CBF modulation was caused by VNS paired with a cognitive task.

### Limitations

This study demonstrated that CBF was modified by paring VNS with a cognitive task. However, we can only speculate that VNS caused such changes by enhancing cortical desynchronization by recruiting the thalamocortical network or that VNS could generate plasticity in the cerebral cortex by repeating the paired stimulation. It is expected that intracranial EEG could verify whether pairing VNS with a cognitive/behavioral task could cause desynchronization in the cerebral cortex.

The 11 channels used in the NIRS measurement were in the bilateral frontal regions and mainly reflected signals from the frontal lobe cortex. Most participants had either wide, multiple, or unknown epileptic foci. Therefore, it is unclear how the epileptic foci distribution affected the blood flow response in the frontal lobe.

## Conclusion

We investigated the immediate effect of VNS on the CBF at rest and during a cognitive task using NIRS. The CBF did not change with VNS stimulation alone, but it increased when the VNS was paired with a cognitive task in a dose-dependent manner in the responder group. It is suggested that VNS amplifies the increase in CBF during cognitive tasks through a mechanism similar to its anti-epileptic effect. This could be an important biological proof of the validity of pairing VNS with rehabilitation.

## Data Availability Statement

The datasets presented in this article are not readily available because participants of this study did not agree for their data to be shared publicly. Requests to access the datasets should be directed to corresponding author (nkunii@nsurg.jp).

## Ethics Statement

The involving human participants were reviewed and approved by the Research Ethics Committee, Graduate School of Medicine and Faculty of Medicine, The University of Tokyo. Written informed consent to participate in this study was provided by the participants’ legal guardian/next of kin.

## Author Contributions

NK, SS, and TK conceptualized the research, and analyzed and interpreted the data. TK and SS performed data curation. NK and TK drafted the manuscript. KK and NS supervised and revised the manuscript. All authors approved the final version of the manuscript.

## Conflict of Interest

NK and SS disclose grant-in-aid from LivaNova PLC for their participation in the Comprehensive Outcomes Registry in Subjects with Epilepsy Treated with Vagus Nerve Stimulation Therapy, independent of this study. KK discloses personal fees from LivaNova Japan and Nihon Kohden Corporation, independent of this study. The remaining authors declare that the research was conducted in the absence of any commercial or financial relationships that could be construed as a potential conflict of interest.

## Publisher’s Note

All claims expressed in this article are solely those of the authors and do not necessarily represent those of their affiliated organizations, or those of the publisher, the editors and the reviewers. Any product that may be evaluated in this article, or claim that may be made by its manufacturer, is not guaranteed or endorsed by the publisher.
