# Supplementary material for: Vagus Nerve Stimulation Amplifies Task-Induced Cerebral Blood Flow Increase
Source: Front Hum Neurosci. 2021 Aug 9;15:726087. doi: 10.3389/fnhum.2021.726087 (PMC8380847; doi:10.3389/fnhum.2021.726087)
Supplement: Supplementary file 1 [file Data_Sheet_1.PDF]

# *Supplementary Material*

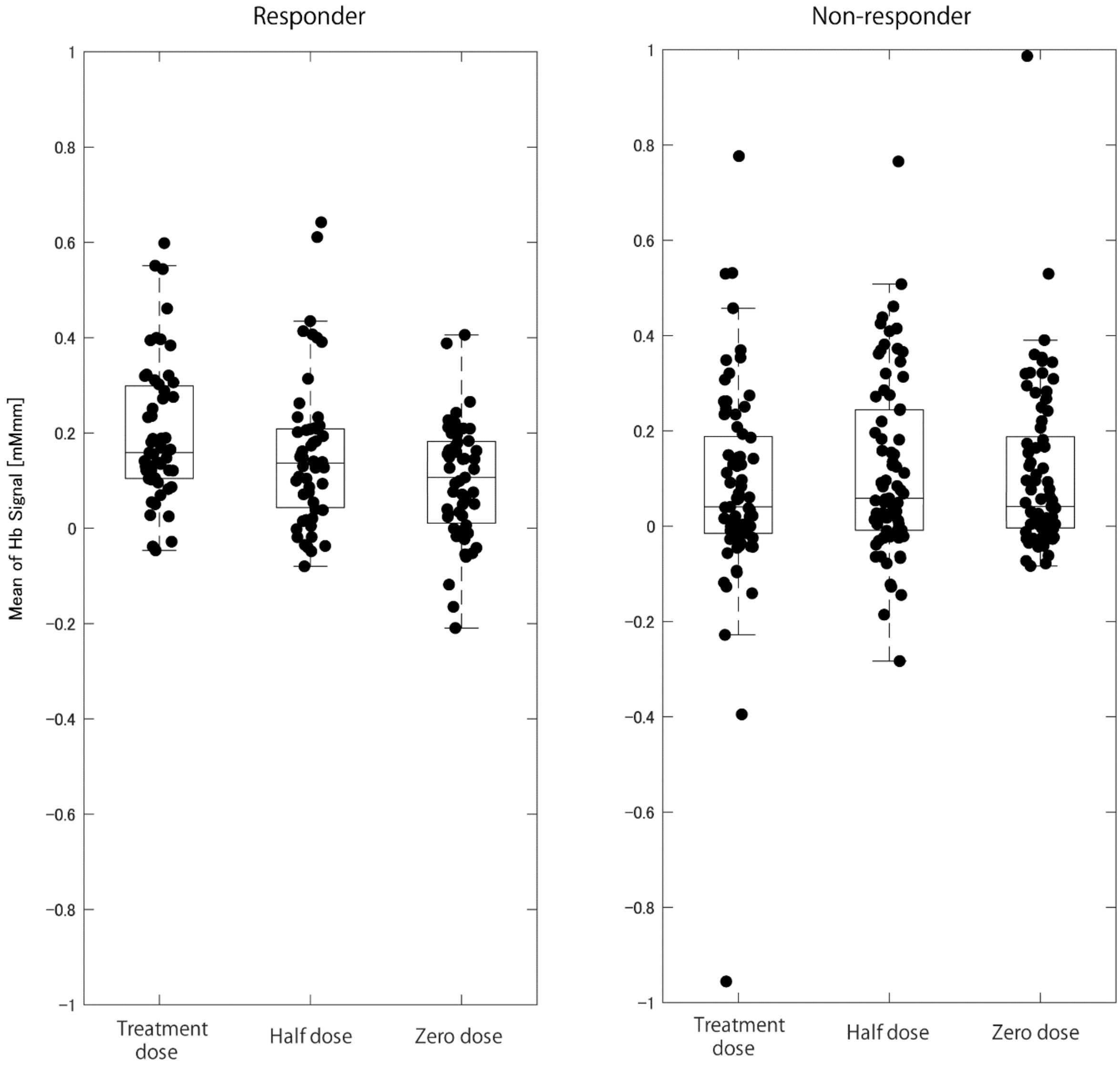


**Supplementary Figure 1.** Box-Whisker plots of mean Hb signal in responders (left) and non-responders (right) during the vagus nerve stimulation at the task condition (treatment, half and zero doses) with individual data points. In the box plots, the bottom and top of each box are the 25th and 75th percentiles of the data, respectively. The line in the middle of each box marks the median. Whiskers above and below the boxes indicate the maximum value within 1.5 times interquartile range over 75th percentile and the minimum value within 1.5 times interquartile range below 25th percentile, respectively.

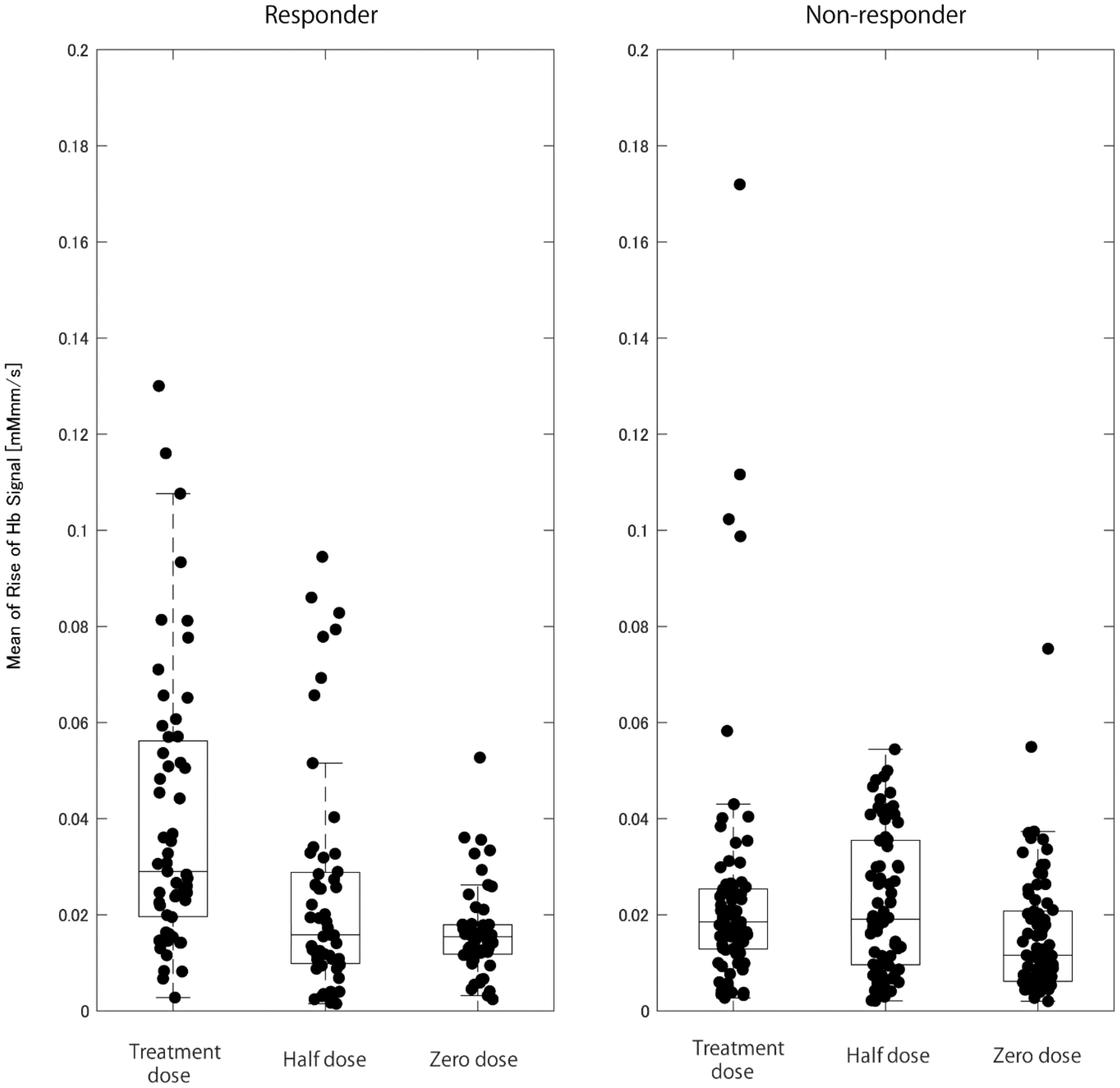


**Supplementary Figure 2.** Box-Whisker plots of mean rise in Hb signal in responders (left) and non-responders (right) during the vagus nerve stimulation at the task condition (treatment, half and zero doses) with individual data points. In the box plots, the bottom and top of each box are the 25th and 75th percentiles of the data, respectively. The line in the middle of each box marks the median. Whiskers above and below the boxes indicate the maximum value within 1.5 times interquartile range over 75th percentile and the minimum value within 1.5 times interquartile range below 25th percentile, respectively.
